# Pulmonary Involvement in Eosinophilic Granulomatosis with Polyangiitis

**DOI:** 10.5334/jbsr.4103

**Published:** 2025-10-24

**Authors:** Elyn Van Snick, Tana Mwewa, Bart Ilsen

**Affiliations:** 1 Vrije Universiteit Brussel, 1050 Ixelles, Belgium

**Keywords:** eosinophilic granulomatosis with polyangiitis, eosinophilia, small vessel vasculitis, airway disease, organizing pneumonia, HRCT

## Abstract

*Teaching point:* Air space opacities, either consolidation or ground glass, are the most frequent imaging finding in EGPA patients with pulmonary involvement, often bilateral and mostly peripheral or random in distribution.

## Case History

**Figure 1:**
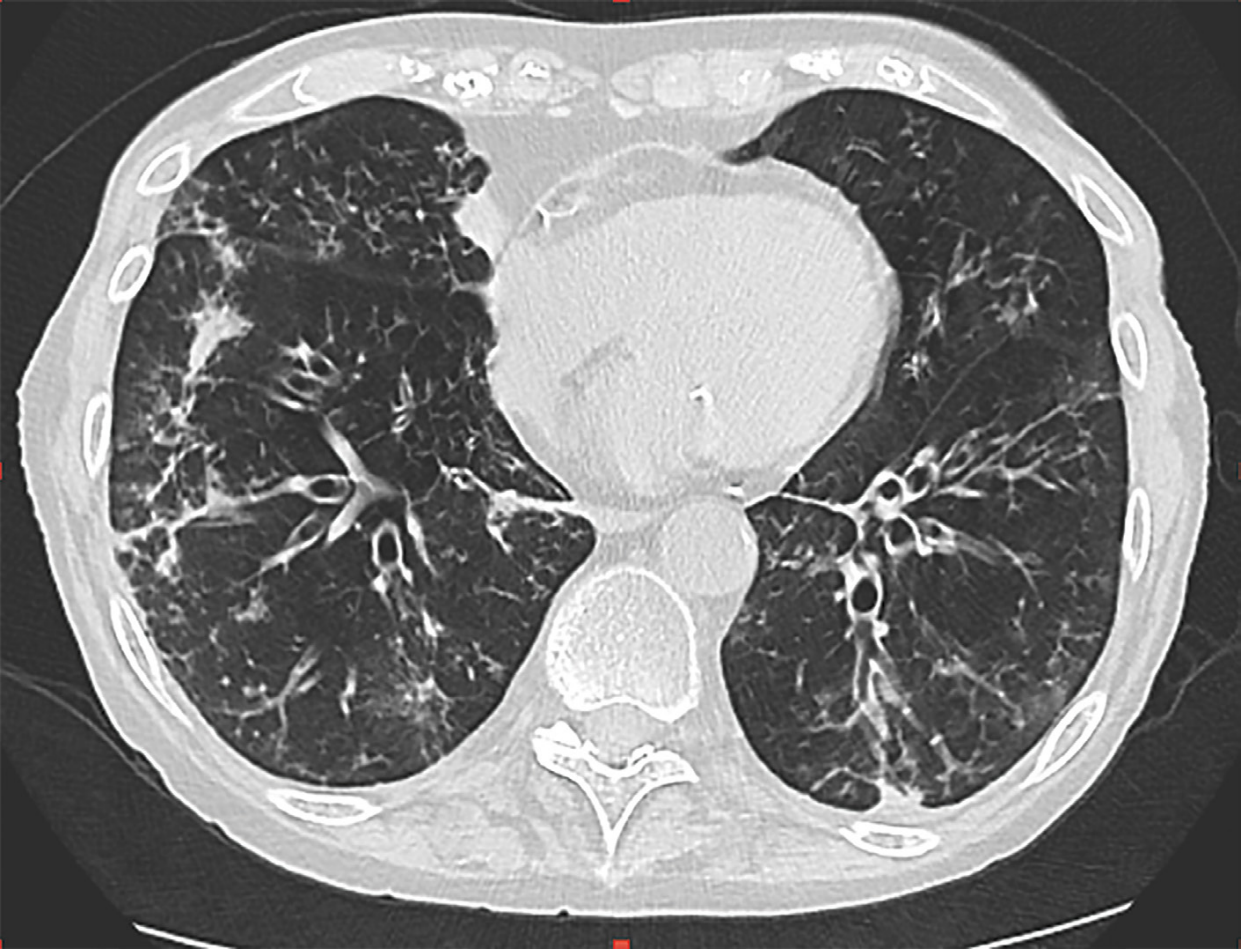
Presence of airway disease with diffuse bronchial wall thickening and cylindrical bronchiectasis in the middle and lower lung areas with peripheral mucus plugging.

**Figure 2:**
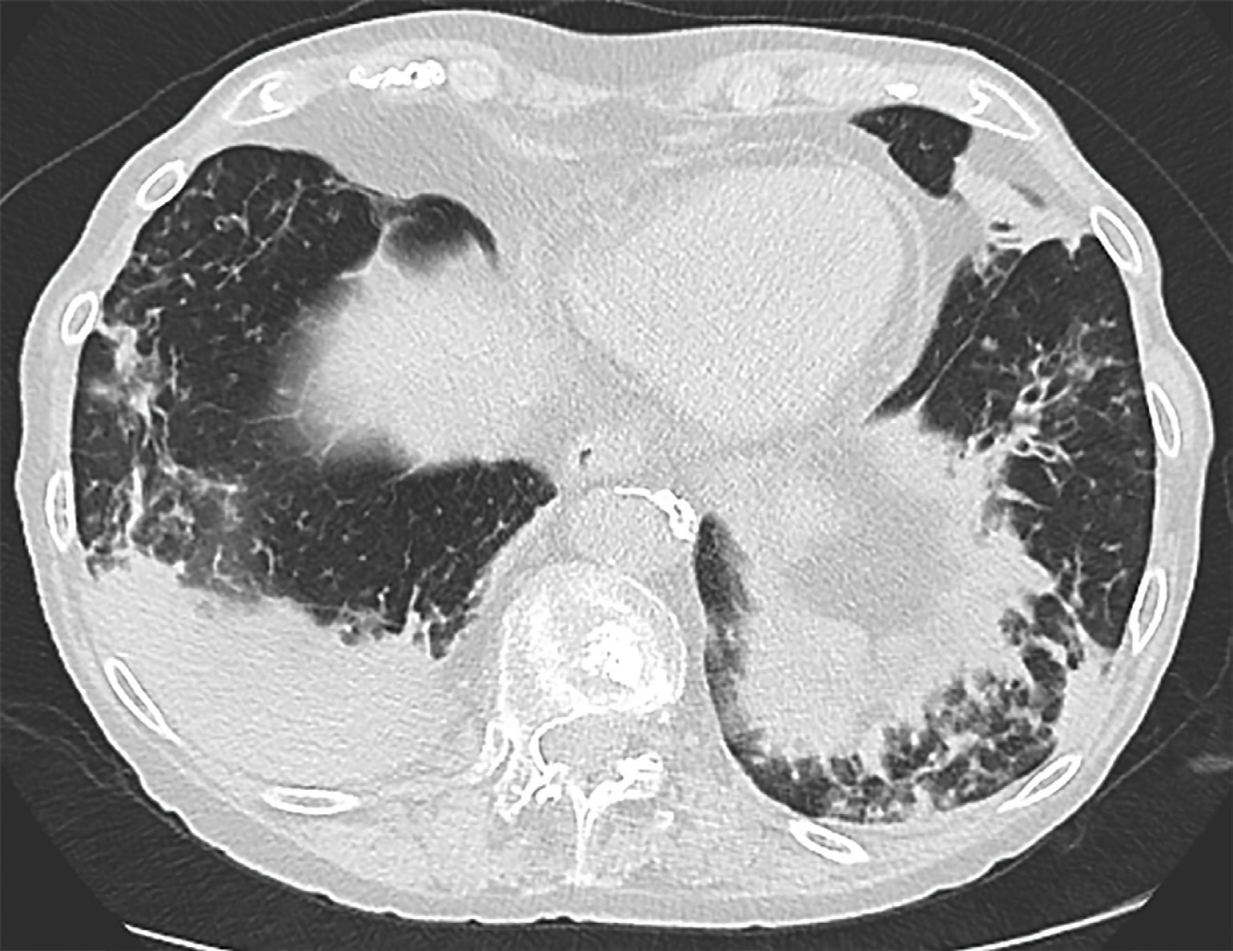
Extensive associated peribronchial and peripherally located consolidations in the middle and lower lung areas.

**Figure 3:**
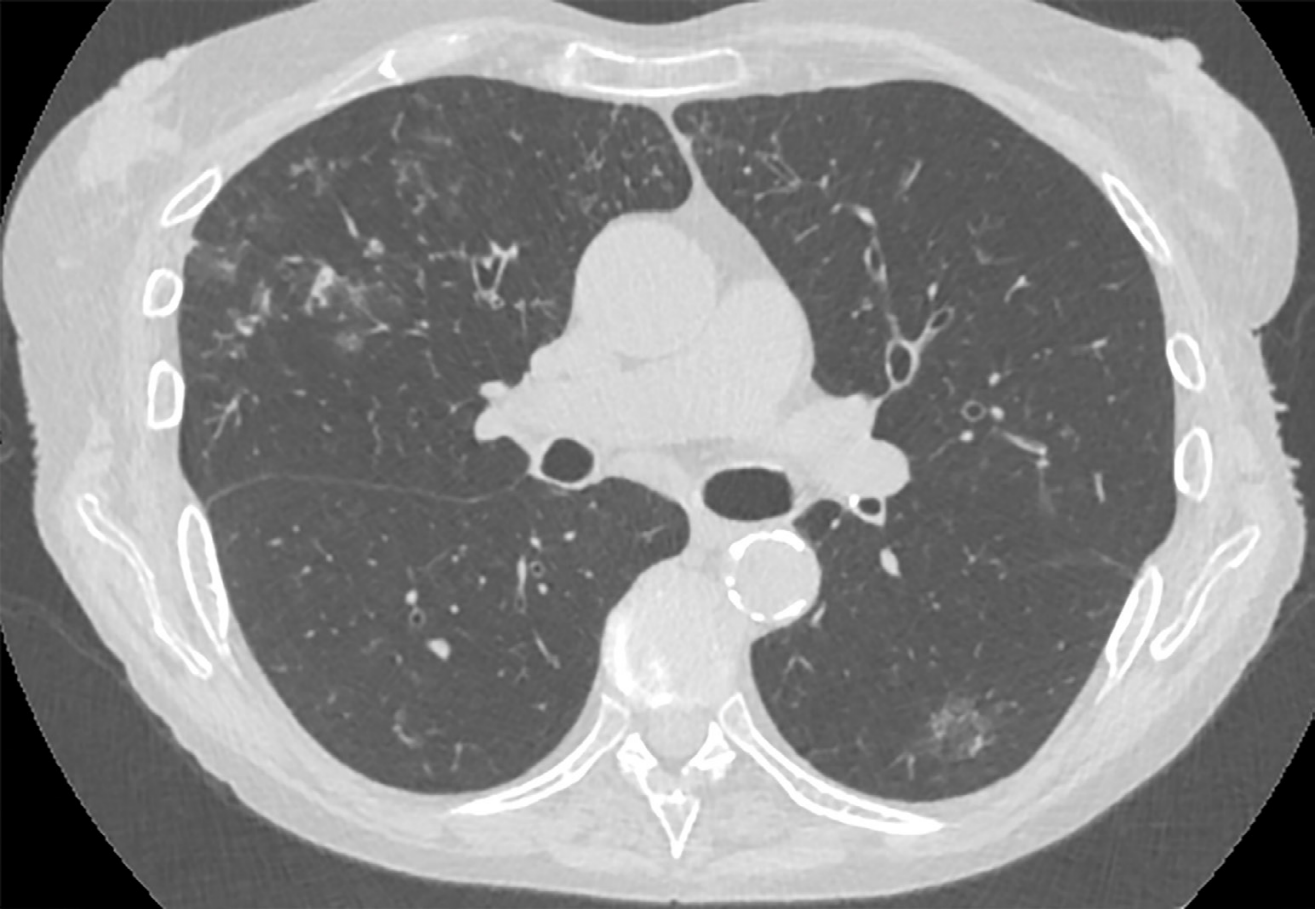
Ill‑defined nodules with centrilobular distributions with associated ground glass opacities.

A 77‑year‑old woman with a known history of eosinophilic granulomatosis with polyangiitis (EGPA) with anti‑neutrophil cytoplasmic antibodies (ANCA) positive status was referred for computed tomography (CT) of the chest because of worsening complaints of dyspnea. CT showed the presence of airway disease with diffuse bronchial wall thickening and cylindrical bronchiectasis in the middle and lower lung areas with peripheral mucus plugging (Figure 1). There were extensive associated peribronchial and peripherally located consolidations in the middle and lower lung areas (Figure 2). In the right upper lobe, ill‑defined nodules with centrilobular distribution were present with associated ground glass opacities (Figure 3).

## Discussion

EGPA, formerly known as Churg–Strauss syndrome, is characterized by the clinical triad of asthma, eosinophilia and a small vessel necrotizing vasculitis. Patients are mostly of middle age and there is no gender predilection. The diagnosis is based on the criteria of the American College of Rheumatology and requires at least four of the following findings: asthma, peripheral eosinophilia, mono‑ or polyneuropathy, transient pulmonary infiltrates, paranasal sinus abnormalities and the presence of extravascular eosinophils on a biopsy specimen. About half of the patients have an ANCA positive status.

The lung is the most commonly involved organ and can manifest as air space opacities, tracheobronchial disease, septal thickening or lung nodules. The most frequent imaging feature is the presence of air space opacities, either consolidation or ground glass, often bilateral, patchy and mostly peripheral or random in distribution. These air space opacities may be transient or migratory. Histologically, the air space disease corresponds to eosinophilic or organizing pneumonia. Another common imaging finding is the presence of bronchial wall thickening and bronchiectasis, which reflects muscle hypertrophy and eosinophilic infiltration of the airway wall. Peribronchial or centrilobular nodules can be seen due to eosinophilic infiltration. Interstitial lung disease with a pattern that resembles usual interstitial pneumonia may be present in a minority of cases. Smooth thickening of the interlobular septa may also be seen, either due to interstitial edema or eosinophilic infiltration of the septa.

Involvement of the heart, as in coronary arteritis, is the main cause for morbidity and mortality in patients with EGPA [1].
